# Cell context-specific expression of primary cilia in the human testis and ciliary coordination of Hedgehog signalling in mouse Leydig cells

**DOI:** 10.1038/srep10364

**Published:** 2015-05-20

**Authors:** Marie Berg Nygaard, Kristian Almstrup, Louise Lindbæk, Søren Tvorup Christensen, Terje Svingen

**Affiliations:** 1 University Department of Growth and Reproduction, Copenhagen University Hospital (Rigshospitalet), Copenhagen DK-2100, Denmark; 2 Department of Biology, University of Copenhagen, Copenhagen DK-2100, Denmark; 3 Department of Toxicology and Risk Assessment, National Food Institute, Technical University of Denmark, Søborg DK-2860, Denmark

## Abstract

Primary cilia are sensory organelles that coordinate numerous cellular signalling pathways during development and adulthood. Defects in ciliary assembly or function lead to a series of developmental disorders and diseases commonly referred to as ciliopathies. Still, little is known about the formation and function of primary cilia in the mammalian testis. Here, we characterized primary cilia in adult human testis and report a constitutive expression of cilia in peritubular myoid cells and a dynamic expression of cilia in differentiating Leydig cells. Primary cilia are generally absent from cells of mature seminiferous epithelium, but present in Sertoli cell-only tubules in Klinefelter syndrome testis. Peritubular cells in atrophic testis produce overly long cilia. Furthermore cultures of growth-arrested immature mouse Leydig cells express primary cilia that are enriched in components of Hedgehog signalling, including Smoothened, Patched-1, and GLI2, which are involved in regulating Leydig cell differentiation. Stimulation of Hedgehog signalling increases the localization of Smoothened to the cilium, which is followed by transactivation of the Hedgehog target genes, *Gli1* and *Ptch1*. Our findings provide new information on the spatiotemporal formation of primary cilia in the testis and show that primary cilia in immature Leydig cells mediate Hedgehog signalling.

The mammalian testis is a complex organ made up of numerous cell types organized into distinct compartments, which are more or less formed by the time of birth (reviewed in 1). The regulation of both testis differentiation and function is complex and involves a series of auto-, juxta-, and paracrine cues that coordinate networks of cellular signalling pathways. These pathways include Hedgehog (HH), Wingless/Int (WNT), Platelet-derived growth factor (PDGF), and Transforming Growth Factor beta/ Bone Morphogenic Protein (TGFβ/BMP) signalling, which all co-operate in a spatiotemporal manner to control both testis organogenesis and function (reviewed in^1^,^2^,^3^,^4^).

A prominent example includes that of Desert HH (DHH) signalling. During fetal development, DHH is produced by Sertoli cells to promote the differentiation of fetal Leydig cells^5^,^6^, and peritubular myoid cells (PMC) during testis cord formation^7^,^8^,^9^. DHH has also been implicated in the regulation of adult Leydig cell specification^8^,^10^, as well as germ cell survival and spermatogenesis^11^. Consequently, aberrant HH signalling is associated with severe disruption in testicular histology, including disorganised seminiferous epithelium, leading to spermatogenic defects and infertility^6^,^7^,^8^,^12^. Moreover, various mutations in *DHH* also cause partial to complete gonadal dysgenesis in humans^13^,^14^,^15^,^16^. Still, much remains to be characterized in order to fully appreciate how HH signalling underpins many of these complex processes of testis development and function. One as yet unappreciated mechanism is that of primary cilia-mediated signal transduction.

Primary cilia are microtubule-based organelles that emanate as solitary, non-motile entities on the cell surface of most vertebrate cell types during growth arrest^17^. They function as unique signalling centres that convey extracellular cues to the inside of cells to control cellular processes during development and in tissue homeostasis. Examples of ciliary signalling pathways include those regulated through Receptor tyrosine kinases (RTKs) and TGFβ receptors, as well as different classes of G-protein-coupled receptors (GPCRs), as in WNT and HH signalling^18^,^19^,^20^,^21^. In the absence of HH ligands, the 12-transmembrane (12TM) receptor, Patched-1 (PTCH1), is localized in the membrane of primary cilia to prevent the ciliary entrance of the 7TM protein Smoothened (SMO). In response to ligand binding, PTCH1 leaves the cilium and SMO enters the ciliary membrane to activate Gli transcription factors (GLI) (reviewed in^22^). Consequently, defects in ciliary assembly or trafficking of signalling components into and out of the ciliary compartment lead to numerous developmental disorders collectively referred to as ciliopathies. These include Bardet-Biedl (BBS), Joubert, and Meckel-Gruber syndromes, as well as Nephronophthisis and polycystic kidney disease (reviewed in^23^). BBS is caused by mutations in genes encoding a series of proteins that form a major protein complex, which controls ciliary assembly and structure as well as sorting of proteins into and out of primary cilia^24^. Interestingly, BBS patients often present with reproductive phenotypes such as Leydig cell or general testis hypoplasia^25^, albeit it is difficult to establish whether these defects arise from a primary failure in testis differentiation or later from disrupted signalling along the adrenal-pituitary-gonadal axis^26^.

Only very few reports have shown the presence of primary cilia in testicular cells, whilst a systematic characterisation in any species during development is lacking. Early electron microscopy studies suggested that Leydig cells in rabbits^27^ and humans^28^,^29^ express primary cilia, and has been corroborated by recent studies on fetal mouse testes also revealing the presence of primary cilia in Leydig cells^30^. Nevertheless, it remains unclear if all, or only a sub-group of Leydig cells form primary cilia and further, at which developmental stage(s) cilia are expressed. Interestingly, testis histology of infertile men with hyperplastic tissue has been shown to display more frequent expression of primary cilia than control tissue^29^, suggesting a developmental regulation in interstitial cells. This is in agreement with reports showing higher frequency of cilia expression by undifferentiated interstitial cells in testes from estrogenised rats^31^ and a prevalence of interstitial cells forming primary cilia in the early stages of mouse fetal testis development^30^.

Some studies have reported on the presence of primary cilia in PMCs^29^,^30^,^31^,^32^. In contrast, cells of the seminiferous epithelium seem to lack primary cilia, although a few ciliated immature Sertoli cells of fetal mouse and prepubertal rat testes have been observed^30^,^32^. Another recent study examining juvenile pig testes reported that a subpopulation of Sertoli cells express a primary cilium in addition to unidentified interstitial cells^33^. Finally, a report examining the ultrastructure of ovarian Sertoli cell tumours indicates the presence of primary cilia in transformed Sertoli cells^34^. Thus it appears, from the animals studied so far, that a subgroup of Leydig cells expresses primary cilia and that PMCs represents the testicular cell type most frequently expressing cilia throughout development. In contrast, Sertoli cells appear to lack a primary cilium at most stages of development, but are reported sporadically in a small number of cells in fetal and postnatal testes. From the very limited available data, human testis seems to mirror this expression profile, albeit human Sertoli cells have thus far not been observed to express primary cilia, with the exception of transformed Sertoli cells of an ovarian Sertoli-cell tumour^34^. As yet we have no knowledge about what signalling pathways are active in primary cilia of the testis or what function they may serve during organogenesis or adulthood.

To further characterise the expression of primary cilia in the human testis, we analysed tissue sections by immunohistochemistry. We found PMCs to be positive for primary cilia, but only a small number of interstitial Leydig cells. Sertoli and germ cells were also largely negative. However, our studies on Klinefelter syndrome testis suggest that immature Sertoli cells express primary cilia. Likewise, less differentiated interstitial cells in hyperplastic testes display a high frequency of primary cilia. We also conducted cell culture experiments showing that HH signalling is regulated by the primary cilium in mouse Leydig cells, which indicate a role for primary cilia in the differentiation of these cells. Our results support the conclusion that primary cilia play a critical role in the development and function of somatic cells of the human testis.

## Results

### Mature Sertoli cells and germ cells of the adult testis do not express primary cilia

**Figure 1:**
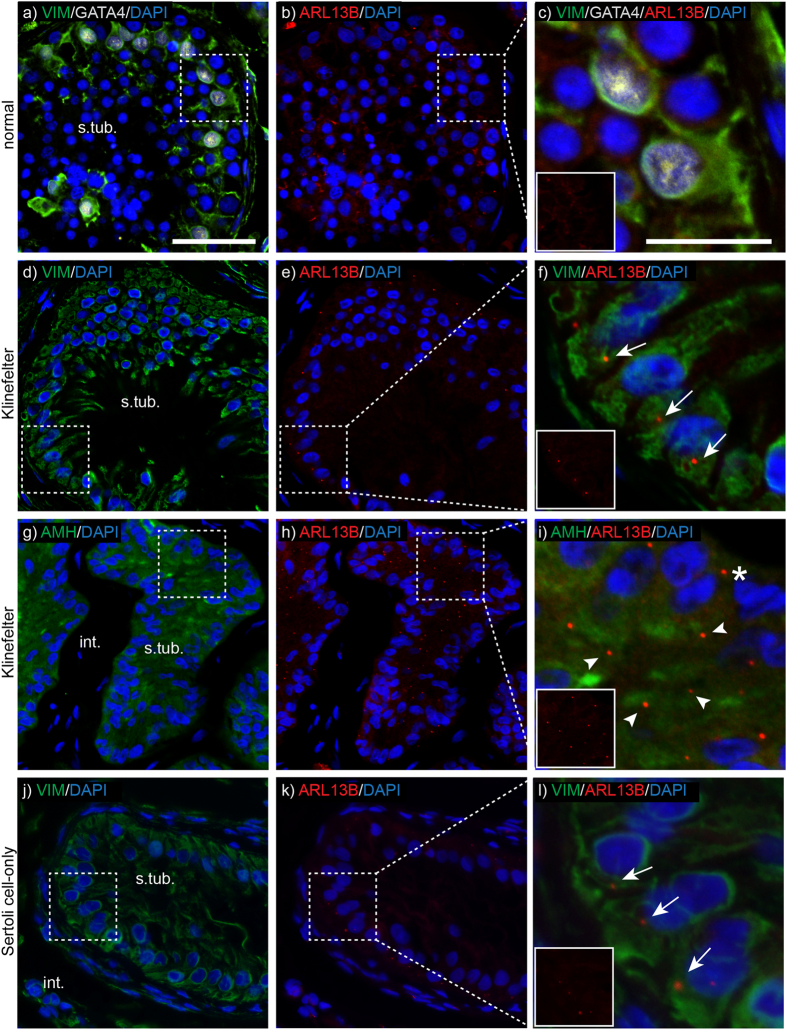
Primary cilia are expressed by immature-like Sertoli cells of Klinefelter testis, but not by mature adult Sertoli cells . **a**-**c**) In adult testis, VIM- (green) and GATA4-positive (white) Sertoli cells do not express ARL13B-positive (red) foci. **d**-**f**) In Klinefelter testis, VIM-positive (green) Sertoli cells of AMH-negative (not shown) seminiferous tubules expresses ARL13B-positive (red) foci that orientate towards the basal membrane (arrows). **g**–**i**) Also AMH-positive (green) Sertoli cells of the Klinefelter testis express ARL13B-positive (red) foci, but orientate most often towards the lumen (arrowheads). Some foci are also orientated towards the basal membrane (asterisk). **j**–**l**) VIM-positive (green) Sertoli cells of Sertoli cell-only tubules were often observed with ARL13B positive foci oriented towards the basal membrane (arrows). Nuclei are stained with DAPI. Insert panels c, f, i, l show red-channel only. *int.* *=* *interstitium; s.tub.* *=* *seminiferous tubule; Scale bars: 50* *μm (two left columns); 20* *μm (right column*).

To characterize the cell-specific expression of primary cilia in the human testis, we performed IHC analysis with the ciliary markers acetylated alpha tubulin (Ac-T) and ADP-ribosylation factor-like 13B (ARL13B), which controls ciliary structure and sensory capacity^35^. Antibodies against Vimentin (VIM) and Gata binding protein 4 (GATA4) were used to demarcate the Sertoli cells. Cells within the seminiferous tubules of normal adult testis (n = 7) typically did not express primary cilia (Fig. 1a–c). On rare occasions, single ARL13B-positive foci were observed in adult Sertoli cells. Spermatogonia at any stage of spermatogenesis did not express primary cilia. The flagellum of spermatids often showed positive staining for Ac-T, but was easily distinguishable from primary cilium by their cellular histology and localisation within the seminiferous tubules.

### Sertoli cells in Klinefelter syndrome and Sertoli cell-only testes express primary cilia in a spatiotemporal manner

To further explore a potential role of primary cilia in Sertoli cell differentiation and/or maturation, testis from a Klinefelter syndrome patient was analysed for the presence of ciliated cells (Suppl. Fig.1 show histology section). The general pool of Sertoli cells were marked using an antibody against VIM, whereas immature Sertoli cells were visualized using an antibody against anti-Müllerian hormone (AMH). The specificity of the AMH antibody was further verified on a fetal human testis at gestational week 21 (GW21; Suppl. Fig. 2). In tubules containing AMH-negative, VIM-positive Sertoli cells, ARL13B-positive foci were observed on most Sertoli cells and almost invariably (>90%) orienting out towards the basement membrane (Fig. 1d–f). In tubules with AMH-positive cells, classified as ‘immature tubules’^36^, the same foci were observed on most Sertoli cells, but now typically oriented towards the lumen (>75%; Fig. 1g–i). In the one fetal testis sample, Sertoli cells typically did not express a primary cilium, although rare single Sertoli cells (<1%) did display ARL13B-positive foci (Suppl. Fig. 2A). ARL13B-positive foci were also observed on Sertoli cells of Sertoli-cell-only testis, typically oriented towards the basement membrane (Fig. 1j–l).

### Peritubular myoid cells (PMCs) of normal and atrophic adult testis express primary cilia

**Figure 2:**
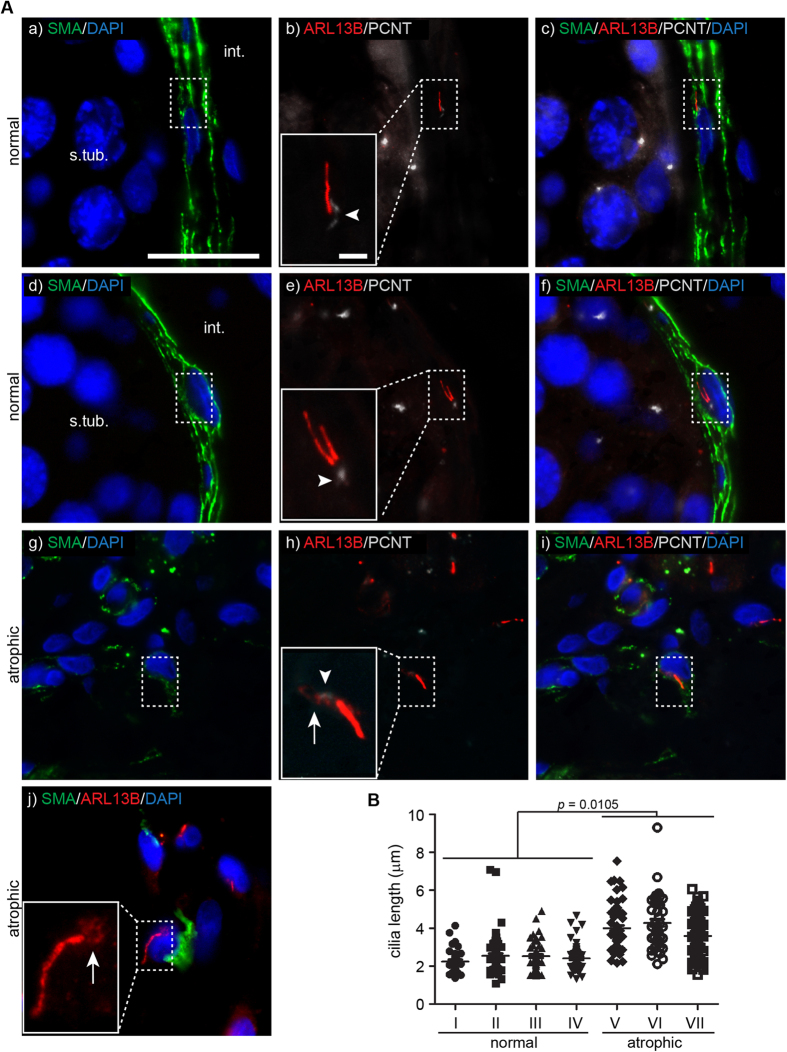
Primary cilia are expressed by peritubular myoid cells (PMCs) in the adult testis and are elongated in interstitial cells of atrophic testis . **A**) **a**-**c**) In adult testis, SMA-positive PMCs (green) lining the seminiferous tubules express primary cilia typically orientating parallel to the basal membrane. The cilium is marked by ARL13B expression (red) and the ciliary pocket by PCNT expression (white; insert: arrowhead). **d**-**f**) Occasionally, the primary cilia of the PMCs occur as pairs (ARL13B-positive cilia; red) extending from a shared ciliary pocket (white; insert: arrowhead). **g**-**j**) In atrophic testis with hyperproliferation, interstitial cells were often positive for SMA (green), cells also maintained primary cilia expression, but additionally the cilium was frequently elongated (j:ARL13B-positive, red). There was also marked presence of ARL13B protein in the PCNT-positive (white) ciliary pocket (h,j: inserts: arrows). Nuclei are stained with DAPI. *int. = interstitium; s.tub. = seminiferous tubule; Scale bars: 20* *μm (all panels); 2* *μm (all inserts*). B) The average length of primary cilia in PMCs of normal testis (I-IV) is significantly shorter than what is observed in myoid-like cells of atrophic testis (V-VII). Each point represents one primary cilia and the mean length within one testis is denoted by a cross-bar. Numerals I-VII represents testicular tissue from individual donors.

Cells of the lamina propria of the seminiferous tubules were frequently observed to express primary cilia. These cells were identified as PMCs by the expression of Smooth-muscle actin alpha 2 (SMA) (Fig. 2A). In normal testis (n = 7), PMCs almost invariably expressed primary cilia (>90%), as identified by ARL13B-stained cilia emerging from PCNT-stained centrosomes (Fig. 2Aa–c). The cilia of PMCs were typically oriented semi-parallel to the basement membrane, with less than 10% observed in a perpendicular orientation, pointing either direction relative to the interstitium. On rare occasions, individual PMCs were observed to express two primary cilia (Fig. 2Ad–f) emanating from a shared ciliary pocket as previously described in other cell types^37^.

We next looked at primary cilia expression in atrophic testis displaying seminiferous tubule hyalinization, as well as peritubular and interstitial fibrosis (n = 5; Suppl. Fig. 1a-c show histology section). The PMCs of the thickened peritubulum were clearly compromised, showing decreased SMA expression and more rounded nuclei than the normal flattened appearance (Fig. 2Ag–j). These cells also formed primary cilia that often were significantly increased in length (Fig. 2Aj). To further substantiate this observation, we performed semi-quantitative measurements from normal (n = 4) and atrophic (n = 3) testis tissues. Only primary cilia observed within the same plane as tissue sections were measured and data represents counts from 10 separate tubuli from the individual biological replicates (Fig. 2B). Although we observed large variability within tissue sections, it was a clear trend (p = 0.0105) towards the primary cilia being elongated in atrophic compared to normal testes. In normal testis, only two primary cilia were measured to exceed 5 μm in length and they were from the same seminiferous tubules of sample II.

### Immature, but not mature Leydig cells express primary cilia in the adult testis

**Figure 3:**
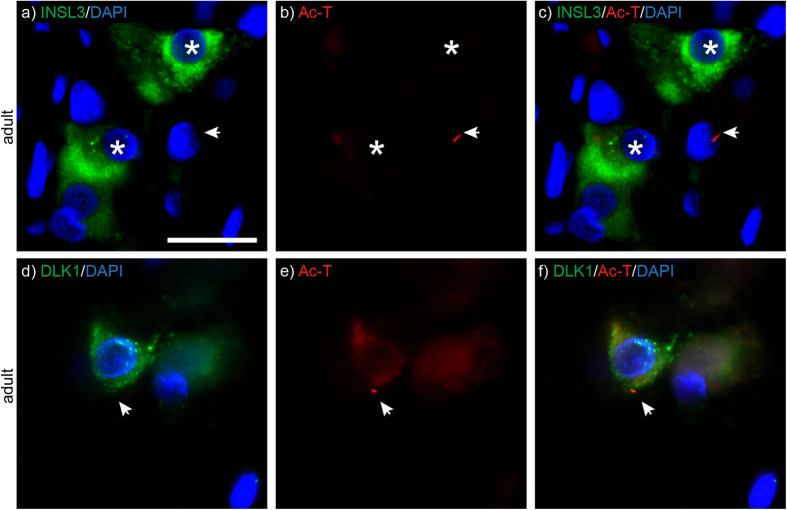
Primary cilia are expressed by a subset of Leydig cells in the adult testis. ** a-c)** In the adult testis, mature Leydig cells expressing INSL3 (green; asterisks) typically do not display a primary cilium, here assessed by Ac-T expression (red; arrowhead). **d-f)** Interstitial cells expressing Ac-T-positive primary cilia (red) often expressed the immature Leydig cell marker DLK1 (green). Nuclei are stained with DAPI*. Scale bar: 20* *μm (all panels).*

We next analysed expression of primary cilia in Leydig cells using antibodies directed against Insulin-like 3 (INSL3) and Delta-like 1 homolog (DLK1), which are expressed by mature and immature Leydig cells, respectively^38^,^39^. Although a large proportion of interstitial cells were found to possess a primary cilium, less than 10% of these were Leydig cells and chiefly when occurring as single cells rather than in clusters of mature Leydig cells (Fig. 3a–c). Most ciliated Leydig cells were DLK1-positive cells (Fig. 3d–f; n = 3), indicating that immature, but not mature Leydig cells express primary cilia.

### Immature mouse TM3 Leydig cells express primary cilia in a cell cycle-dependent manner

**Figure 4:**
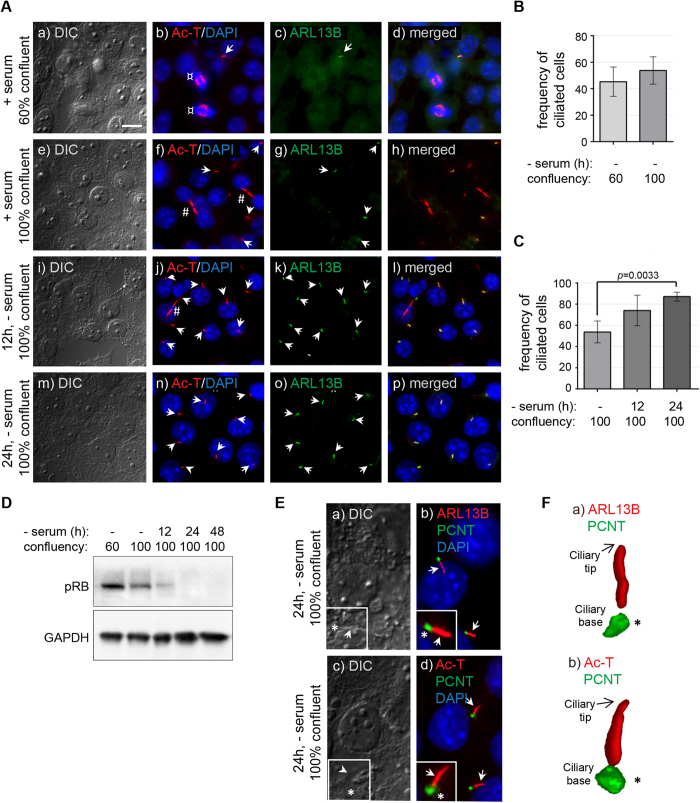
Characterization of primary cilia formation in TM3 mouse Leydig cells. ** A)** TM3 cells were visualized by differential interference contrast (DIC) microscopy. Primary cilia were detected using antibodies against Ac-T (red; arrows) and ARL13B (green; arrows), both localizing to the cilium. Ac-T also localized to mitotic spindles (b; ¤) and mid-bodies (f, j; #). When cultured in the presence of serum, TM3 cells infrequently expressed a primary cilium (**a-d**), with a small, but noticeable rise in frequency when reaching 100% confluency (**e-h**). When followed by serum depletion to induce growth arrest, the frequency of cells expressing a primary cilium increased significantly after 12 h (**i-l**) and 24 h (**m-p**). *Nuclei were stained with DAPI. Scale bar* *=* *10* *μm.* **B-C)** Frequency of ciliated TM3 cells in culture estimated after manual counting of cells. **B)** When cultured in the presence of serum, a small increase in cilia frequency is observed as the cells become confluent. **C)** Following serum deprivation, the frequency of ciliated cells increases within 12h and reaches close to 90% by 24 h. *p-value was determined by one-way ANOVA.* **D)** Western blot analysis showing expression of phosphorylated RB (pRB) decreasing as TM3 cells grow from low (60%) to high (100%) confluency, and gradual loss of expression following serum deprivation of confluent cells (100%) measured at 12, 24 and 48 h. GAPDH served as loading control. **E)** To further verify the presence of intact primary cilia, the ciliary markers (arrows) ARL13B (**b;** red) and Ac-T (**d;** red) were co-stained with the centrosomal marker PCNT (**b,d;** green) for detection of the ciliary base. Inserts show the cilium (arrow) emerging from the ciliary base (asterisk). *Nuclei were stained with DAPI.* **F)** 3D-rendering of primary cilia emerging from the ciliary base (asterisk), marked using ARL13B (**a;** red), Ac-T (**b;** red) and PCNT2 (**a-b;** green) staining of TM3 cells.

Hedgehog (HH) signalling is known to be required for Leydig cell differentiation, thus we explored a putative relationship between cilia-mediated HH signalling and Leydig cell development. We used cultures of the immature mouse Leydig cell line TM3, to investigate whether primary cilia in these cell types are associated with working HH signalling machinery. In order to define the cell system and to optimize the formation of primary cilia, cells were initially cultured at two different cell densities as well as in serum-depleted medium for different time points to induce growth arrest. This was followed by immunocytochemical (ICC) analysis with different markers for primary cilia, including ARL13B and Ac-T. Withdrawal of serum was associated with a reduced number of cells in mitosis and cytokinesis concomitantly with an increased number of cells forming primary cilia (Fig. 4A). Thus, the frequency of cells forming primary cilia in cultures with 100% confluency increased from 54% at 0h of serum depletion to about 75% and 85% of cells in cultures that were serum depleted for 12 and 24 h, respectively (Fig. 4B,C). WB analysis confirmed that the increase in the frequency of ciliated cells during serum depletion was associated with growth arrest, as evidenced by decreased phosphorylation of Retinoblastoma protein (pRb), which marks cycling cells (Fig. 4D).

In order to evaluate the specificity of the antibodies and further assess the base and tip of primary cilia on TM3 cells after 24 h of serum depletion, cilia were co-stained for PCTN to mark the ciliary base (Fig. 4E). Additional light microscopy also enabled visualization of the cilium and the ciliary base region, which appeared as a prominent structure in DIC analysis and co-localized with the centrosomal markers (indicated with asterisks in Fig. 4E). These structures were further studied by 3D reconstructed images, confirming the unique positioning of the ciliary base and tip (Fig. 4F). Subsequent ICC analysis was therefore combined with DIC in order to determine the position of the base and the tip of the primary cilia.

### TM3 Leydig cells express primary cilia with working Hh signalling

**Figure 5:**
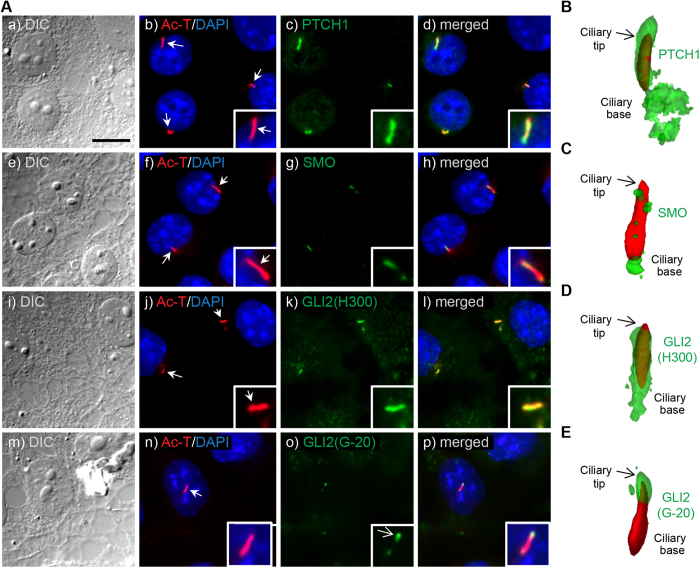
Hedgehog components localize to the primary cilium of TM3 cells. **** TM3 cells were serum-starved for 24 h before immunofluorescence analysis. **A)** Cells were visualized by DIC microscopy and nuclei stained with DAPI. **a-d)** PTCH1 (green) and the ciliary marker Ac-T (red) co-localized at the cilium (**d;** merged, insert), but with PTCH1 displaying a broader expression pattern also localizing to the ciliary base. **e-h)** SMO (green) displayed patchy expression both at the cilium (red) and ciliary base (**h;** merged, insert). GLI2 (green) was assessed by two antibodies (H300 and G-20), with **i-l)** GLI2 (H300) showing expression along the entire cilium (**l;** merged, insert), and **m-p)** GLI2 (G-20) only at the ciliary tip (**p;** merged, insert). *Scale bar: 10* *μm (all panels).* **B-E)** 3D-rendering showing the localization of PTCH1, SMO and GLI2 (green) of primary cilia, axoneme marked with Ac-T (red) with ciliary base immediately below.

To analyse if HH signalling components are present and functional in primary cilia of TM3 cells, we initially performed ICC analysis (Fig. 5A) in combination with 3D reconstruction imaging (Fig. 5B-E), showing that PTCH1, SMO, and GLI2 all localize to primary cilia of growth arrested cells. GLI2 expression was shown with two different antibodies marking either the entire length of the cilium (H-300) or most prominently the ciliary tip (G-20). Further, PTCH1 was highly enriched both along the cilium and at the ciliary base region, whereas SMO showed only weak localization to the cilium, indicating a low level of HH signalling in the cells under these culture conditions.

**Figure 6:**
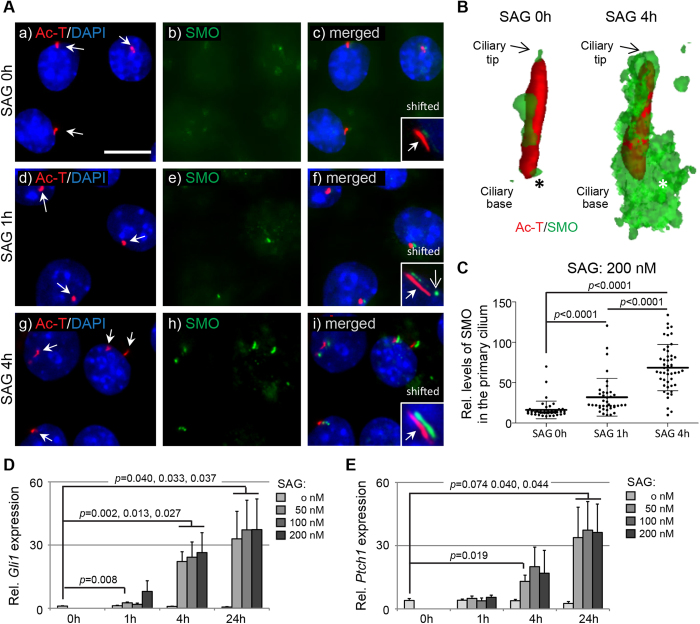
Activation of Hedgehog pathway recruits SMO to the primary cilium in TM3 Leydig cells. **** TM3 cells were cultured in the presence of the SMO agonist (SAG) to monitor response to HH activation. **A)** In control cells (**a-c;** 0 h), primary cilia marked by Ac-T (red; arrows) only displayed occasional, speckled appearance of SMO (green). After 1 h in the presence of 100 nM SAG (**d-f**), a stronger and more frequent localization of SMO to the primary cilia was observed, now also at the ciliary base (**f;** insert; arrow), and this was even more apparent after 4 h of SAG-stimulation (**g-i***). Nuceli were stained with DAPI. Scale bar = 10* *μm*. **B)** 3D-rendering revealed a significant recruitment of SMO (green) to the primary cilium, both at the axoneme (Ac-T-positive; red) and the base (asterisk) following SAG-stimulation for 1 and 4 h, relative to 0 h. **C)** Relative fluorescence intensities of SMO in primary cilia of TM3 cells cultured in the presence of 100 nM SAG and measured at 0, 1 and 4 h. A significant increase in ciliary SMO was observed after both 1 and 4 hours of SAG stimulation*. P-values were determined by one-way ANOVA.* **D-E)** qRT-PCR measurements of relative *Gli1* and *Ptch1* mRNA abundance following SAG stimulation. **D)** Already at 1 h there is a quantifiable elevation of *Gli1* mRNA, which was significant at all measured concentrations after 4 h of stimulation, with a further upregualtion at 24 h. There was no significant difference in mRNA levels between 50, 100 and 200 nM SAG at 4 and 24 h. **E)** *Ptch1* mRNA levels were elevated following 4 h SAG stimulation, with a further upreguation after 24 h*. p-values were determined by two-tailed (unpaired) Student’s t-test.*

Next, TM3 cells were stimulated with the SMO-agonist SAG for 1 and 4 hours in growth arrested cells to determine if a functional HH machinery is present in the cilium. As indicated by ICC analysis, control cells (0 hours) showed low levels of ciliary SMO with only occasional punctuated appearance along the cilium as evidenced in 3D reconstructed images (Fig. 6A). Following stimulation with SAG (100 nM) for 1 hour there was an increase of SMO localization to the cilium, which was more pronounced after 4 hours of SAG stimulation (Fig. 6A). 3D reconstructed images of the cilium clearly revealed a dramatic increase in the accumulation of SMO along the length of the cilium, as well as at the lower part of the cilium, indicating docking of vesicular SMO at the ciliary base for transport of SMO into the cilium (Fig. 6B). Relative quantification of SMO levels at the primary cilium on TM3 cells revealed significant, and about 2- and 5-fold increases in the level of ciliary SMO after 1 and 4 hours of SAG stimulation relative to unstimulated cells (Fig. 6C). To further verify the activation of HH signalling in the ciliated TM3 cells, we finally performed qRT-PCR assays to measure the relative mRNA expression of the HH target genes, *Gli1* and *Ptch1*. TM3 cells were grown in the presence of 0, 50, 100 and 200 nM SAG for 0, 1, 4 and 24 hours before harvest and RNA extraction. The mRNA levels of both *Gli1* (Fig. 6D) and *Ptch1* (Fig. 6E) were upregulated in a time- and dose-dependent manner, with activation of *Gli1* being most significant.

## Discussion

It is well established that HH, PDGF, TGFβ/BMP and WNT signalling critically regulate testis development and function. These signalling pathways were previously shown to be regulated by the primary cilium in various cell types, but it remains unknown if primary cilia are necessary for testis development and function. Here, we have characterized in detail the cell-specific expression of primary cilia in the adult human testis and show that i) Sertoli and germ cells typically do not express primary cilia in the adult testis, ii) PMCs constitutively express primary cilia; and iii) immature, but not fully differentiated Leydig and Sertoli cells possess primary cilia. Further, immature-like Leydig cells in culture were shown to possess a functioning HH signalling machinery.

Although Sertoli cells of the adult human testis do not express primary cilia, ARL13B-positive foci were occasionally associated with Sertoli cells. It was recently shown that ciliary membranes can become associated with the mother centriole following disassembly and internalization of the primary cilium before mitosis^40^. Thus, we speculate that the few ARL13B-positive foci seen in the tubular epithelium represent remnant ciliary membrane at the centriole of mitotic Sertoli cells. If so, it also implies that less mature Sertoli cells possess primary cilia prior to disassembly, which seems to be the case from more recent studies. For instance, a subset of immature Sertoli cells of the fetal mouse testis express primary cilia^30^, which was also observed in a human fetal testis (Suppl. Fig. 2). Sertoli cells of juvenile rat and pig testes also express primary cilia^32^,^33^ and further, a gradual decrease in frequency of ciliated Sertoli cells from juvenile stages to puberty was demonstrated in pig testis^33^.

In the absence of juvenile human testis tissues, we analysed Sertoli cells of adult Klinefelter syndrome testis to gain some initial insight into a putative temporal regulation of ciliary formation in these cell types. Here, seminiferous tubules are typically void of germ cells and classified according to Sertoli cell morphology as mature type A or immature type B^36^, with the latter also expressing AMH, a functional marker for immature Sertoli cells. Interestingly, we found Sertoli cells of both types of SCO tubules to possess distinct foci positive for the ciliary marker ARL13B. In AMH-negative SCO tubules, these foci typically oriented towards the basement membrane, whereas those of AMH-positive SCO tubules often oriented towards the lumen. This could suggest a transitional phase of Sertoli cell organisation within the seminiferous tubules prior to the onset of spermatogenesis. In Klinefelter syndrome testes however, there is a gradual loss of germ cells and tubular degeneration from fetal life into adulthood^41^, hence these immature-like Sertoli cells are not necessarily Sertoli cells that have failed to mature properly, but can also represent a dedifferentiated phenotype. Additionally, we cannot rule out a mechanism in which it is the loss of germ cells that trigger abnormal assembly of primary cilia. Indeed, when tubules are devoid of germ cells, as in the SCO testis, we observed foci positive for the ciliary marker ARL13B in Sertoli cells, again most frequently orientated towards the basal membrane.

PMCs are strategically located at the interface between the interstitium and the tubular epithelium which allows them to transduce signals between most cells types of the testis, and they are known to influence Leydig and Sertoli cells^42^. Whether primary cilia of PMCs mediate any of these signals remain unknown. Still the constitutive expression of cilia by PMCs observed both by us and others^29^,^30^,^31^,^32^ make it plausible. Therefore, future studies should focus on characterizing the receptor complement of the ciliary membrane to delineate what signalling pathways may be involved. Another possibility is that the cilia on PMCs can function as mechanical sensors, as these cells are both embedded in extracellular matrix and capable of rhythmic contractions^43^. This is further suggested by their phenotypic similarities with vascular smooth muscle cells (VSMCs), where primary cilia have been shown to function as mechanochemical sensors responding to both mechanical pressure and ECM proteins^44^.

We also observed a significant increase in ciliary length on PMCs in atrophic testes. This pathology – often observed in infertile men – is typified by fibrosis of both the peritubular and interstitial compartments, as well as hyalinisation of seminiferous tubules. Peritubular fibrosis involves thickening of the tubular wall through cellular hypertrophy and deposition of ECM, but also involves phenotypic changes to PMCs such as altered marker protein expression and nuclear morphology^42^,^45^,^46^,^47^. It remains unclear why primary cilia are elongated in this pathology, but one possibility is that it is an inflammatory response. An increase in inflammatory cells is well documented in the atrophic testis (reviewed by^42^ and increased ciliary length has been reported to occur in response to inflammation in other tissues^48^,^49^. Yet, ciliary length is also influenced by numerous other factors^50^, all of which can be responsible for the observed elongation in the PMCs.

In most mammals, the testis contains two sequential populations of Leydig cells; fetal Leydig cells that disappear after birth, and adult Leydig cells that replaces the diminished fetal population (reviewed in^1^,^51^). Whether these cell populations arise from distinct cell lineages or share a common precursor remains a topic of debate, although recent studies in mice suggest that the latter is the case^10^. Either way, it is commonly thought that Leydig cells do not proliferate by mitotic division, but rather are recruited from a pool of precursor cells^52^,^53^. And one of the key molecules involved in Leydig cell recruitment is Sertoli cell-derived DHH, which act on PTCH receptors on the precursor cells^5^,^6^,^8^,^9^,^54^,^55^.

In the adult human testis, we found that mature Leydig cells expressing INSL3 did not possess primary cilia, whereas less differentiated (DLK1-positive, INSL3-negative) Leydig cells often did. We speculated that ciliary HH signalling in precursor cells could contribute to Leydig cell differentiation and hence studied the immature Leydig cell line TM3. These cells were found to express primary cilia at a high rate upon serum starvation and also to express HH signalling components on the cilium. PTCH1, which is expressed by most undifferentiated interstitial cells during testis development^6^, also localized to the cilium. Following HH activation, SMO was recruited to the cilium, ultimately resulting in transcriptional activation of the target genes *Gli1* and *Ptch1*. These results clearly reveal the potential of Leydig cells, at least TM3 cells, to mediate HH signalling via the primary cilium.

Since primary cilia were expressed by DLK1-positive, but not INSL3-positive Leydig cells in the human testis, it strongly suggests that Leydig cells lose primary cilia as they differentiate into a mature phenotype. It may also suggest a further involvement of primary cilia in Leydig cell development. It is well established that DHH signalling induces both fetal and adult Leydig cell differentiation in the mouse testis^5^,^6^,^8^,^9^,^54^,^55^, and that functional *DHH* mutations in humans lead to disrupted testis development^13^,^14^,^15^,^16^. We propose that primary cilia are involved in the regulation of Leydig cell recruitment or differentiation by mediating signalling by paracrine factors such as DHH.

In summary, only somatic cells of the adult human testis were found to express primary cilia, and expression seems to depend on the developmental stage of Leydig and Sertoli cells. In Leydig cells, primary cilia were found to be responsive to HH signalling, which is known to be crucial for proper gonadal development. Spermatogenesis is highly dependent on both Leydig and Sertoli cells, and our results may indicate an important role for primary cilia in sperm production by ensuring proper development early in life. This notion is strengthened by observations in patients with cilia defects, for instance BBS patients whom often present with compromised fertility and Leydig cell, or general testis hypoplasia^25^. Thus, disruptions in primary cilia signalling may have greater implications for male sexual development and reproductive health than anticipated so far.

## Materials and Methods

### Ethical considerations

Tissues used in this study were obtained from the human tissue archives at the Department of Growth and Reproduction (Rigshospitalet). Written informed consent from patients, tissue handling and experiments were carried out in accordance with the protocols approved by the regional Danish Committee for Medical Research Ethics (Permit number: H-1-2012-007).

### Human tissues

Human tissue samples were obtained from orchidectomised testes or testicular biopsies performed in relation to testicular tumours. Patients were 22–46 years of age, and diagnosed with either an overt tumour (non-seminoma or seminoma) or carcinoma in situ (CIS) without any tumour present. The normal non-malignant testis tissues were obtained either from adjacent to a tumour or from the contralateral testis. The Klinefelter syndrome patient (25 years of age) was diagnosed with Leydig cell tumour. Samples included in this study were; normal testis (n = 7), Klinefelter syndrome testis (n = 1), atrophic testis (n = 5) and SCO testis (n = 2). The fetal (GW21) testis sample was obtained from a medical abortion collected for diagnostic purposes and donated for research. Tissue samples were fixed overnight at 4 °C in buffered formalin or 4% paraformaldehyde, dehydrated and embedded in paraffin wax.

### Cell culture and stimulation of HH signalling

Mouse immature Leydig cells (line TM3;^56^ were cultured in minimum essential medium DMEM containing 10% FBS (Invitrogen) at 37 °C, 5% CO_2_, and 95% humidity. Cells were passaged before reaching confluency at ~1.6 × 10^5^ cell/cm^2^ by adding 1% trypsin (Invitrogen) in phosphate-buffered saline (PBS) to detach cells, followed by resuspension in culture medium and transfer into new tissue culture flasks. Formation of primary cilia was verified by immunofluorescence (IF) analysis in cell cultures at different cell densities and in cultures deprived of serum for 0, 12, 24 and 48 h as previously described^57^. In some experiments, cells were serum starved for 24 h and subsequently stimulated with 50, 100 and 200 nM of N-Methyl-N’-(3-chlorobenzo[b]thiophene-2-carbonyl)-1,4-diaminocyclohexane (SAG), which functions as a SMO agonist that activate HH signalling (Calbiochem).

### SDS-PAGE and Western blot (WB) analysis

SDS-PAGE and WB analyses were carried out essentially as described previously^58^. Briefly, cell lysates were prepared in 0.1% SDS lysis buffer followed by sonication and measurement of protein concentrations on a BioDrop. Proteins were separated by SDS-PAGE using 10% Bis-Tris precast gels (Invitrogen) followed by electrophoretic transfer to nitrocellulose membranes (Invitrogen). Membranes were incubated for 1 h at room temperature in blocking buffer (5% skim milk in Tris-buffered saline (TBST); 10 mM Tris–HCl (pH 7.5), 120 mM NaCl, 0.1% Tween 20) before incubation with primary antibodies overnight at 4 °C. Membranes were washed then incubated with horse radish peroxidise (HRP)-conjugated secondary antibodies for 1 h at room temperature. Washed membranes were developed with Enhanced Chemiluminescence detection system (Amersham Bioesciences ECL) and images captured using a Chemidoc Imaging System (Bio-Rad).

### Immunohistochemistry (IHC)

IHC experiments were carried out on 4 μm sections mounted on glass plates essentially as described previously^59^. In brief, tissue sections were dewaxed in Tissue-Tek Tissue-Clear (Sakura), washed in 100% ethanol and rehydrated through a graded ethanol-water series. Antigen retrieval was performed by heat treatment for 15 min (microwave) immersed in TEG buffer (pH 9.0), then cooled for 15 min at room temperature. Slides were washed in TBS and pre-blocked in 5% bovine serum albumin (BSA) in TBS at room temperature for 30 min. Primary antibodies (Table 1) in blocking solution were added to slides in humidity chambers and left overnight at 4 °C. Slides were washed in TBS and incubated with fluorochrome-conjugated secondary antibodies for 1 h in the dark. Slides were counterstained with 4,6-diamidino-2-phenylindole (DAPI; Sigma) before washed and mounted with ProLong Gold anti-fade reagent (Life Technologies). Due to auto-fluorescence by mature Leydig cells containing high levels of lipofucin, some IF experiments were carried out with an additional incubation step in 0.1% Sudan Black-B solution (Sigma) for 30 min immediately after secondary antibody removal, then washed in TBS 3 × 10 min before mounting. Secondary antibodies were used at 1:600 dilutions and were: donkey anti-mouse Alexa-488, -568, -647, donkey anti-rabbit Alexa-488, -568, -647, and donkey anti-goat Alexa-488, -568 (Life Technologies).

### Immunocytochemistry (ICC)

ICC experiments were carried out as previously described^57^. Briefly, cells were cultured on 12-mm sterile HCl-cleansed coverslips, washed in ice-cold PBS and fixed with 4% PFA for 15 min at room temperature. Cells were washed in PBS and permeabilised with 0.2% Triton X-100 and 1% BSA in PBS for 12 min followed by incubation in blocking buffer (2% BSA in PBS) for 30 min at room temperature or overnight at 4 °C before transfer to a humidity chamber. Coverslips were subsequently incubated with primary antibodies (Table 1) in blocking buffer for 90 min at room temperature followed by wash in blocking buffer and incubation for 45 min with fluorochrome-conjugated secondary antibodies (see IHC). Coverslips were washed in blocking buffer and briefly incubated with DAPI for nuclear staining, followed by PBS washes, then mounted on microscope slides in anti-fade mounting solution. Cilia frequency was determined by quantifying the number of ciliated and non-ciliated cells of a minimum number of 100 cells for each sample in at least three replicates.

### Immunofluorescence (IF) microscopy and 3D imaging analysis

Tissue sections and cell cultures were imaged with an Olympus BX-61 microscope and captured using cellSens Dimensions V1.6 software (Olympus Ltd.). 3D reconstructed images were created from z-stacks using add-on features of the cellSens software. Further image processing was performed with Adobe Photoshop version CS6. To quantify the relative fluorescence of SMO in the primary cilium (Fig. 4C), a region of interest (ROI) was first drawn along the periphery of the structure (Suppl. Fig. 2). The mean fluorescence intensity was then measured within the selected region and data represents the mean of >30 cilia counted in three biological replicates for each treatment. The data were tested for statistical significance using one-way analysis of variance (ANOVA).

### qRT-PCR analysis

Total RNA was extracted from cell pellets using the NucleoSpin RNA II purification kit (Macherey-Nagel) following the manufacturer’s recommendations. cDNA was synthesised from 1 μg total RNA in the presence of random hexamers/oligo-dT and AMV reverse transcriptase (Affymetrix, USB Products) as described elsewhere^60^. qRT-PCR assays were performed with SYBR green (Agilent Technologies) on a Stratagene Mx3000P QPCR System in 20 μl reactions essentially as described previously^60^. All primer pairs (Table 2) were designed to span intron-exon boundaries (except *Rn18s*), amplify products <150 nucleotides, their specificity verified by sequencing and efficiency determined to be 90–105%. Relative transcript abundance was calculated by the comparative Ct (2^−ΔCt^) method, using the geometric mean of *Rn18s* and *Rps29* expression for normalization of Ct-values.

## Additional Information

**How to cite this article**: Nygaard, M.B. *et al.* Cell context-specific expression of primary cilia in the human testis and ciliary coordination of Hedgehog signalling in mouse Leydig cells. *Sci. Rep.* **5**, 10364; doi: 10.1038/srep10364 (2015).

## Supplementary Material

Supplementary Information

## Figures and Tables

**Table 1:** List of antibodies and working dilutions.

**Antibody**	**Origin**	**Dilution**	**Cat. No.**	**Source**
Ac-T	Mouse	1:3000	T7451	Sigma-Aldrich
ARL13B	Rabbit	1:500	17711-1-AP	Proteintech
AMH	Mouse	1:150	-	Gift from R. Cate
DLK1	Rabbit	1:500	-	Gift from C.H. Jensen
GATA4	Goat	1:250	Sc-1237	Santa Cruz
GLI2 (H-300)	Rabbit	1:100	Sc-28674	Santa Cruz
GLI2 (G-20)	Goat	1:100	Sc-20291	Santa Cruz
INSL3	Rabbit	1:1500	HPA028615	Atlas Antibodies
PCNT	Goat	1:200	Sc-28145	Santa Cruz
PTCH1	Rabbit	1:100	Sc-9016	Santa Cruz
SMA	Mouse	1:2500	7817-500	Abcam
SMO	Rabbit	1:500	AB7817	Abcam
VIM	Mouse	1:3000	Sc-73717	Santa Cruz

**Table 2:** List of primers used for qRT-PCR assays.

**Gene**	**Accession No.**	**Primer sequence (5’ – 3’)**	**Size (bp)**
*Rn18s*	NR_003278	F: GATCCATTGGAGGGCAAGTCT	103
		R: CCAAGATCCAACTACGAGCTTTTT	
*Rps29*	NM_009093	F: TGAAGGCAAGATGGGTCAC	127
		R: GCACATGTTCAGCCCGTATT	
*Gli1*	NM_010296	F: ATCCCACAGGCACACAGGA	66
		R: CTCCTCTCTCTCCAGGTCCTC	
*Ptch1*	NM_008957	F: TGACAAAGCCGACTACATGC	61
		R: GTACTCGATGGGCTCTGCTG	
