# Supplementary material for: Cell context-specific expression of primary cilia in the human testis and ciliary coordination of Hedgehog signalling in mouse Leydig cells
Source: Sci Rep. 2015 May 20;5:10364. doi: 10.1038/srep10364 (PMC4438617; doi:10.1038/srep10364)
Supplement: Supplementary Information [file srep10364-s1.doc]

Supplementary Files for manuscript:

**Cell context-specific expression of primary cilia in the human testis and ciliary coordination of Hedgehog signaling in mouse Leydig cells**

Marie Berg Nygaard^1,2^, Kristian Almstrup^1^, Louise Lindbæk^2^, Søren Tvorup Christensen^2^ and Terje Svingen^1,3,*^

1. *University Department of Growth and Reproduction, Copenhagen University Hospital (Rigshospitalet), Copenhagen DK-2100, Denmark*
2. *Department of Biology, University of Copenhagen, Copenhagen DK-2100, Denmark*
3. *Current address.*

*Department of Toxicology and Risk Assessment, National Food Institute, Technical University of Denmark, Søborg DK-2860, Denmark*

* Correspondence to: [tesv@food.dtu.dk](mailto:tesv@food.dtu.dk)

Running Title: Expression of primary cilia in the human testis and Hh-mediated Leydig cell differentiation

SUPPLEMENTARY FIGURES


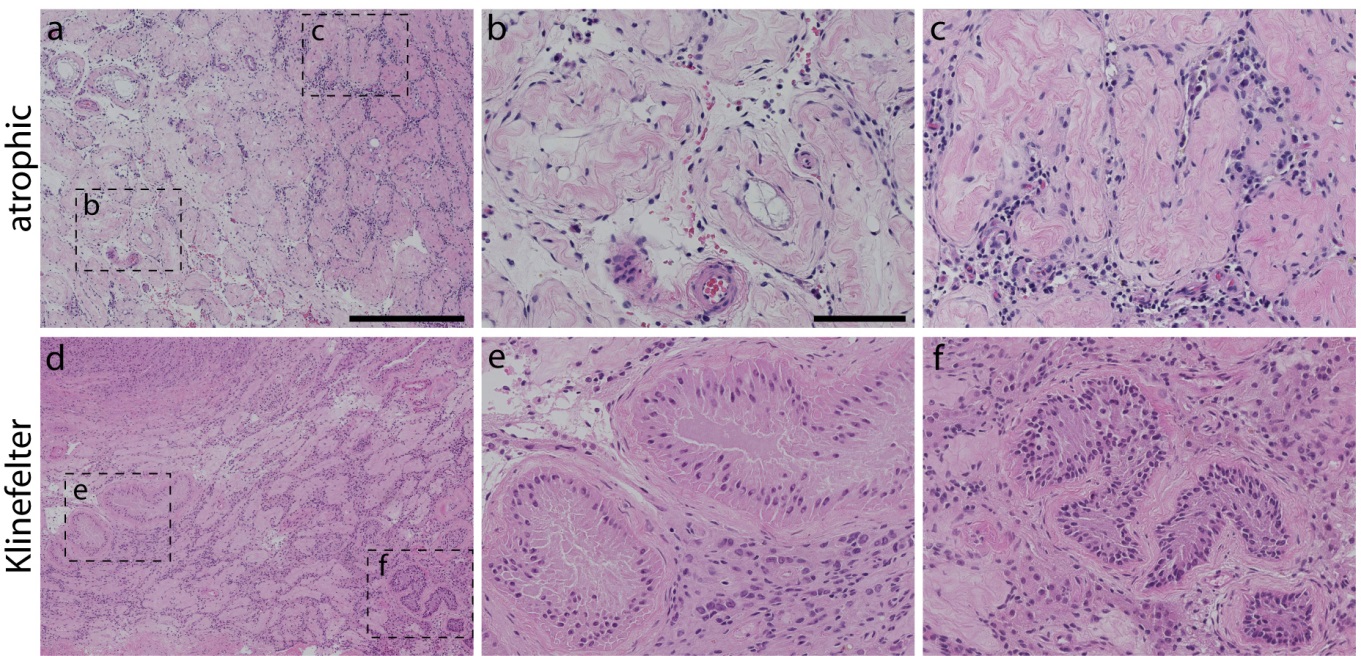


**Suppl. Fig. S1: Histology sections of atrophic and Klinefelter testis. a)** Haematoxylin and Eosin (H&E) staining of atrophic testis. **b,c)** Magnified regions of atrophic tissue with hyaline deposits. Degenerated seminiferous tubule remnants remain discernable. **d)** H&E staining of Klinefelter testis. **e)** Magnified region of mature type A tubules and **f)** immature type B tubules. *Scale bars: 500µm (left columns); 100 µm (two right columns*).


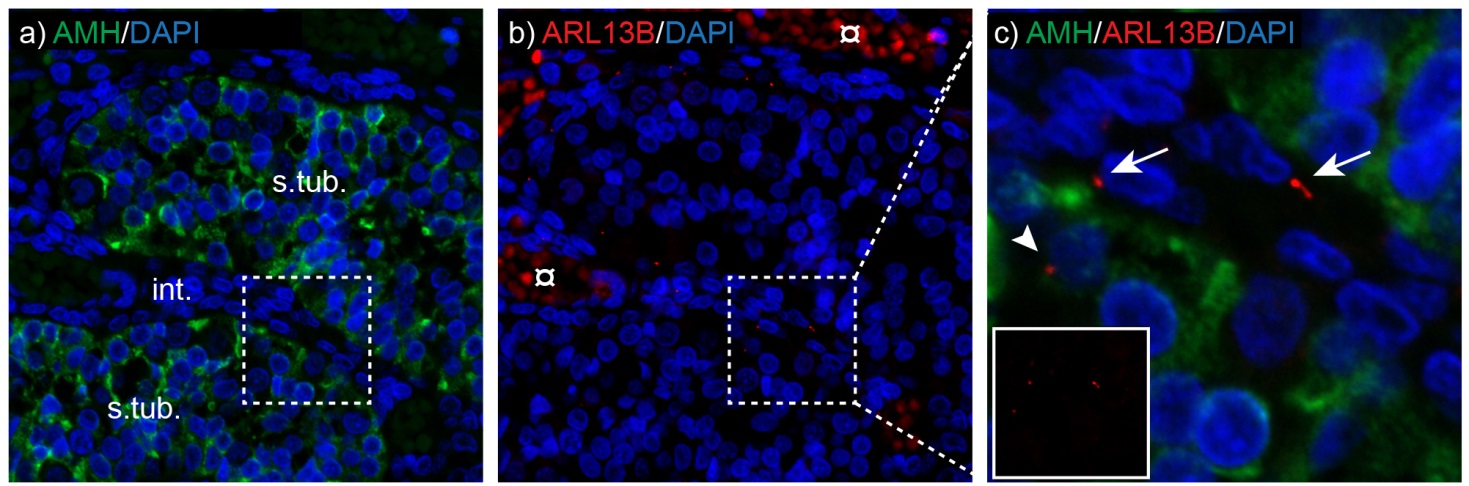


**Suppl. Fig. S2: Primary cilia are expressed by a subset of interstitial cells in human fetal testis.** **a-c)** In fetal testis at GW21, AMH-positive (green) Sertoli cells do typically not express primary cilia, apart from a rare few (<1%) displaying ARL13B-positive (red) foci. A proportion of interstitial cells (<20%) do express primary cilia at this developmental stage. Nuclei are stained with DAPI. Insert panel (c) show red-channel only. int.= interstitium; s.tub.=seminiferous tubule; ¤ demarcates blood cells. Scale bars: 50µm (two left columns); 20 µm (right column).


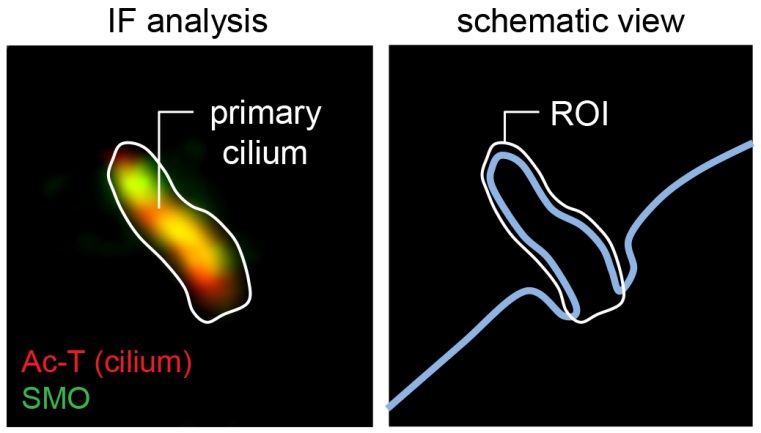


**Suppl. Fig. S3: Schematics of method for the quantification of SMO (green) fluorescence in the primary cilium.** A region of interest (ROI) was drawn along the primary cilium stained with a marker, e.g. acetylated alpha-Tubulin (Ac-T; red). The mean fluorescence intensity was measured from the defined region (dotted white line).
